# RNAget: an API to securely retrieve RNA quantifications

**DOI:** 10.1093/bioinformatics/btad126

**Published:** 2023-03-10

**Authors:** Sean Upchurch, Emilio Palumbo, Jeremy Adams, David Bujold, Guillaume Bourque, Jared Nedzel, Keenan Graham, Meenakshi S Kagda, Pedro Assis, Benjamin Hitz, Emilio Righi, Roderic Guigó, Barbara J Wold, GA4GH RNA-Seq Task Team, Jeremy Adams, Alvis Brazma, David Bujold, Julia Burchard, Joe Capka, Michael Cherry, Laura Clarke, Brian Craft, Manolis Dermitzakis, Mark Diekhans, John Dursi, Michael Sean Fitzsimons, Zac Flaming, Romina Garrido, Alfred Gil, Paul Godden, Matt Green, Roderic Guigo, Mitch Guttman, Brian Haas, Max Haeussler, Benjamin Hitz, Bo Li, Sten Linnarsson, Adam Lipski, David Liu, Simonne Longerich, David Lougheed, Jonathan Manning, John Marioni, Christopher Meyer, Stephen Montgomery, Alyssa Morrow, Alfonso Munoz-Power Fuentes, Jared Nedzel, David Nguyen, Kevin Osborn, Francis Ouellette, Emilio Palumbo, Irene Papatheodorou, Dmitri Pervouchine, Arun Ramani, Jordi Rambla, Bashir Sadjad, David Steinberg, Jeremiah Talkar, Timothy Tickle, Kathy Tzeng, Sean Upchurch, Saman Vaisipour, Sean Watford, Barbara Wold, Zhenyu Zhang, Jing Zhu

**Affiliations:** Biology and Biomedical Engineering, California Institute of Technology, Pasadena, CA 91125, United States; Centre for Genomic Regulation (CRG), The Barcelona Institute of Science and Technology, Barcelona, Catalonia 08003, Spain; Ontario Institute for Cancer Research, Toronto, ON M5G 0A3, Canada; Department of Human Genetics, McGill University, Montreal, QC H3A 0G4, Canada; Department of Human Genetics, McGill University, Montreal, QC H3A 0G4, Canada; Broad Institute, Cambridge, MA 02142, United States; Department of Genetics, Stanford University, Stanford, CA 94305, United States; Department of Genetics, Stanford University, Stanford, CA 94305, United States; Department of Genetics, Stanford University, Stanford, CA 94305, United States; Department of Genetics, Stanford University, Stanford, CA 94305, United States; Centre for Genomic Regulation (CRG), The Barcelona Institute of Science and Technology, Barcelona, Catalonia 08003, Spain; Centre for Genomic Regulation (CRG), The Barcelona Institute of Science and Technology, Barcelona, Catalonia 08003, Spain; Department of Medicine and Life Sciences, Universitat Pompeu Fabra (UPF), Barcelona, Catalonia 08002, Spain; Biology and Biomedical Engineering, California Institute of Technology, Pasadena, CA 91125, United States

## Abstract

**Summary:**

Large-scale sharing of genomic quantification data requires standardized access interfaces. In this Global Alliance for Genomics and Health project, we developed RNAget, an API for secure access to genomic quantification data in matrix form. RNAget provides for slicing matrices to extract desired subsets of data and is applicable to all expression matrix-format data, including RNA sequencing and microarrays. Further, it generalizes to quantification matrices of other sequence-based genomics such as ATAC-seq and ChIP-seq.

**Availability and implementation:**

https://ga4gh-rnaseq.github.io/schema/docs/index.html.

## 1 Introduction

Gene expression quantification by sequencing [e.g. RNA sequencing (RNA-seq) and single-cell RNA-seq] or by hybridization (e.g. microarrays) is the major contemporary research tools for phenotyping human cells and tissues. Translating these methods and datatypes into clinical medicine and routine healthcare is a natural progression (Haque et al. 2017, https://doi.org/10.1186/s13073-017-0467-4), following the trajectory of whole-exome and whole-genome DNA sequencing (Birney et al. 2017, https://www.biorxiv.org/content/early/2017/10/15/203554). Rapidly increasing RNA data published worldwide present compelling opportunities for large-scale data mining from multiple sources. For instance, the European Genome-phenome Archive, the database of Genotypes and Phenotypes, the Encyclopedia of DNA Elements (ENCODE), the Genotype-Tissue Expression project (GTEx), the National Institute of Health Genomic Data Commons, the International Human Epigenome Consortium (IHEC) among others have established large repositories intended for sharing. Yet there are unmet challenges for handling huge numbers of files from diverse sources, coupled with limitations arising from jurisdictional and consent restrictions on data access. A goal for the field is a federated model in which users can mine and combine data from diverse sources that include centralized repositories (biobanks, national or regional healthcare providers, and commercial clouds) as well as individual laboratories or clinics. To help realize this vision, the Global Alliance for Genomics and Health (GA4GH) develops and maintains a suite of interoperable standards (Birney et al. 2017).

RNA-seq produces a single quantitative value for expression level from a gene or transcript isoform (Mortazavi et al. 2008, https://doi.org/10.138/nmeth.1226) and resulting quantifications are typically stored and provided as individual files for bulk RNA from a given tissue sample. This data-type has now been joined by much larger sparse matrix files for RNAs detected in each of millions of individual cells or nuclei comprising a sample (reviewed in Haque et al. 2017). Several formats for storing quantification matrices that readily manage bulk or single-cell data, such as loom (https://loompy.org), annData (Wolf et al. 2018, https://doi.org/10.1186/s13059-017-1382-0), hdf5 (https://www.hdfgroup.org/HDF5/), and matrix market (https://math.nist.gov/MatrixMarket/) meet all needs. A standardized API for the delivery of quantification data from all experimental types and data formats is therefore needed for interoperability and data sharing.

Here, we introduce RNAget, an open standard for secure retrieval of expression quantifications drawn from multiple individual samples that is applicable to microarray data and RNAseq from bulk, pseudobulk single-cell or single-cell data. This protocol allows a client to retrieve matrices containing data from multiple samples, uses existing community data formats, and provides an option for matrix slicing. RNAget is a part of a family of compatible GA4GH protocols designed to enable efficient and secure discovery and exchange of many types of primary and derived genomic data (Rehm et al. 2021, https://doi.org/10.1016/j.xgen.2021.100029).

## 2 Results

### 2.1 Schematic of protocol

**Figure 1. Schematic of RNAget protocol:**
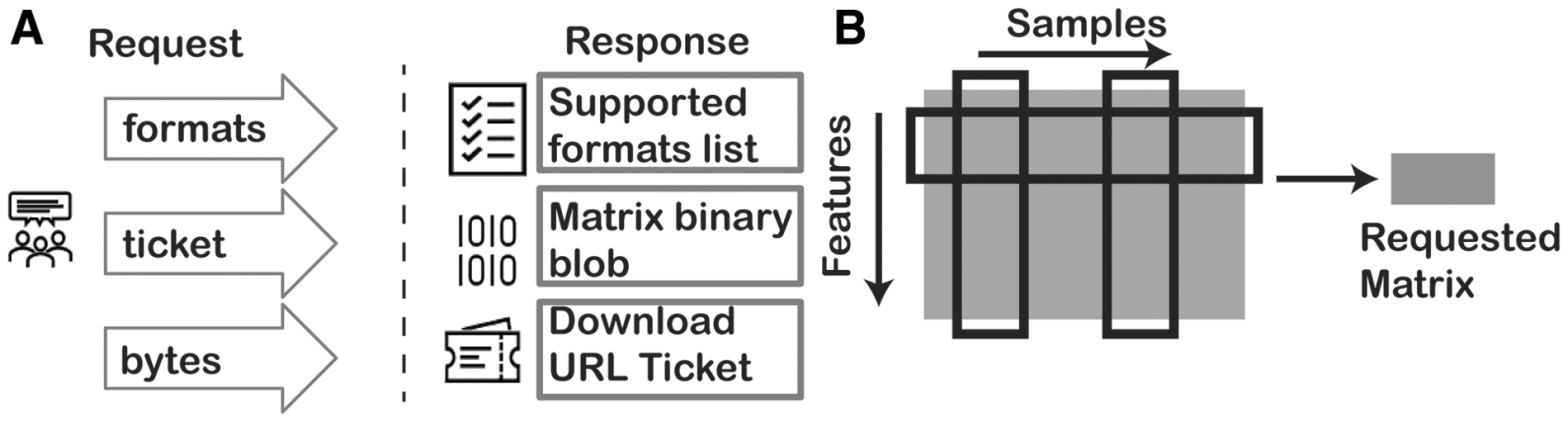


The client creates an HTTP request to a URL (determined via another discovery service) with a transfer format. Requests for quantifications are made to one of two sets of HTTPS endpoints. One endpoint of the set returns a small JSON block with a URL for the client to download the data, the other returns the requested data as an inline blob (Fig. 1A). Unlike conventional file download, an RNAget request can optionally retrieve a slice of the original matrix by including filters on the samples and/or genomic features (Fig. 1B). This brings the potential to greatly improve performance and focus data mining by limiting retrieval to a desired subset of the data.

### 2.2 Security

RNAget is designed to retrieve quantifications from both fully open genetic data sources (e.g. ENCODE) and data subject to increased security and authorization requirements (e.g. controlled access human data). Sensitive information transmitted on public networks must be protected using Transport Level Security (TLS). RNAget can therefore be integrated into existing authorization and authentication infrastructure that use the OAuth2.0 protocol (https://tools.ietf.org/html/rfc6749) to authorize data requests.

### 2.3 Initial implementations

RNAget has now been implemented by several large-scale data providers including ENCODE, GTEx, the Canadian Distributed Infrastructure for Genomics, and IHEC. The user interface for searching the ENCODE implementation is https://www.encodeproject.org/rnaget-report/?type=RNAExpression and the direct API endpoints are https://rnaget.encodeproject.org/service-info. The API is a Python/Flask application using Elasticsearch as a storage and filtering backend. The user interface for the GTEx implementation is https://gtexportal.org/rnaget/docs and the direct API endpoints are https://gtexportal.org/rnaget/service-info. The API is a Python/FastAPI using Loom files as a storage backend. The IHEC Data Portal delivers hd5 matrices of epigenomic datasets selected using the portal’s filtering tools (Bujold et al. 2016, https://doi.org/10.1016/j.cels.2016.10.019). Examples of client code can be found at https://github.com/ga4gh-rnaseq/schema/blob/master/README.md.

## 3 Discussion

The RNAget API standard defines requests for delivery of processed RNA data for either bulk RNA samples or contemporary single-cell data. To reduce the time and effort to implement, it recommends existing and widely used file formats for transport given above and it allows data providers to use any internal data storage model. RNAget is designed to securely transport both open and controlled access data, applying security measures (TLS, HTTPS, and OAuth 2.0) as essential components of the specification.

The RNAget API describes a set of endpoints for retrieval of quantification data such as feature level expression data from RNA-seq type assays and signal data over a range of genome base coordinates from epigenomic experiments. While the initial focus of this standard was to handle RNA-seq and other transcriptome quantifications, the concept adapts for ChIP-seq, ATAC-seq, and other types of epigenomic ‘counting’ assays as shown by IHEC.

The matrix structure is designed to work well with dataframes. The ability to slice the data matrix makes it easier to merge data from multiple sources retrieved with a given filter or set of filters, thus reducing the total volume of data to download. Implementors can define additional slicing filters within the API. For example, a provider of single-cell resources could implement an additional slicing filter on a specific cell type (or types) to apply in tandem with a slicing filter on particular genes of interest. More generally, the RNAget API can make it easier to write software to compare, co-mingle, and analyze data retrieved from multiple and potentially geographically dispersed servers.

## References

[btad126-B1] Birney E , Vamathevan J, Goodhand P. Genomics in healthcare: GA4GH looks to 2022. bioRxiv 203554. 2017.

[btad126-B2] Bujold D , Morais D, Gauthier C et al The International Human Epigenome Consortium data portal. Cell Syst 2016;3:496–9.e2. 27863956 10.1016/j.cels.2016.10.019

[btad126-B3] Haque A , Engel J, Teichmann SA et al A practical guide to single-cell RNA-sequencing for biomedical research and clinical applications. Genome Med 2017;9:75. 28821273 10.1186/s13073-017-0467-4 PMC5561556

[btad126-B4] Mortazavi A , Williams B, McCue K et al Mapping and quantifying mammalian transcriptomes by RNA-Seq. Nat Methods 2008;5:621–8. 18516045 10.1038/nmeth.1226 PMC13303166

[btad126-B5] Rehm HL , Page AJ, Smith L et al GA4GH: International policies and standards for data sharing across genomic research and healthcare. Cell Genomics 2021;1:100029. 35072136 10.1016/j.xgen.2021.100029 PMC8774288

[btad126-B6] Wolf F , Angerer P, Theis F. SCANPY: Large-scale single-cell gene expression data analysis. Genome Biol 2018;19:15. 29409532 10.1186/s13059-017-1382-0 PMC5802054

